# Supplementary figures and images for: Structural insights into the atypical filament assembly of pyrin domain-containing IFI16
Source: EMBO J. 2025 Nov 5;44(24):7702–20. doi: 10.1038/s44318-025-00626-7 (PMC12705702; doi:10.1038/s44318-025-00626-7)

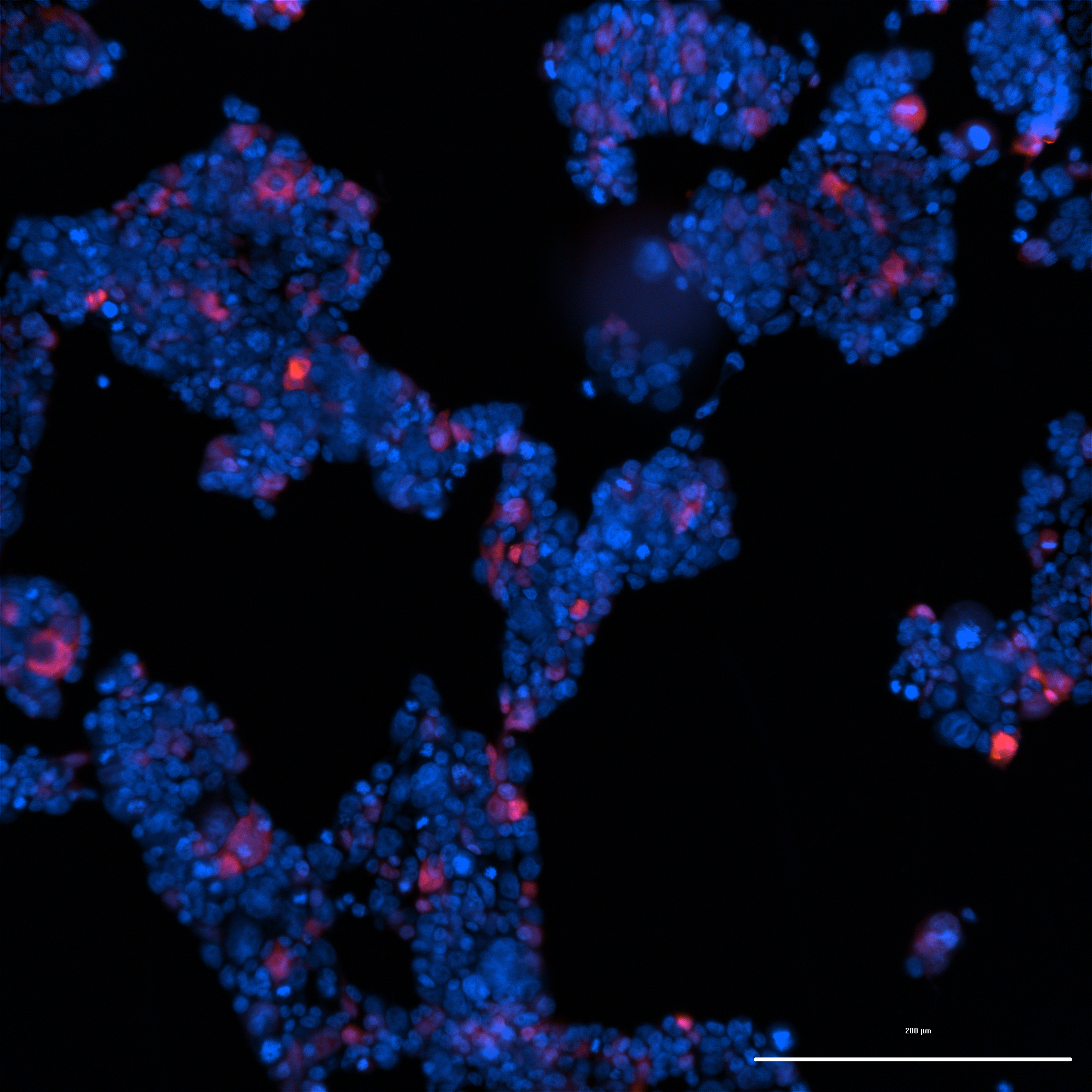

Supplement: Supplementary file 3 — Source data Fig. 3 [file 44318_2025_626_MOESM3_ESM.zip › Source data for Figure 3/3D/D71K_IFI16PYD_20x_2m.png]

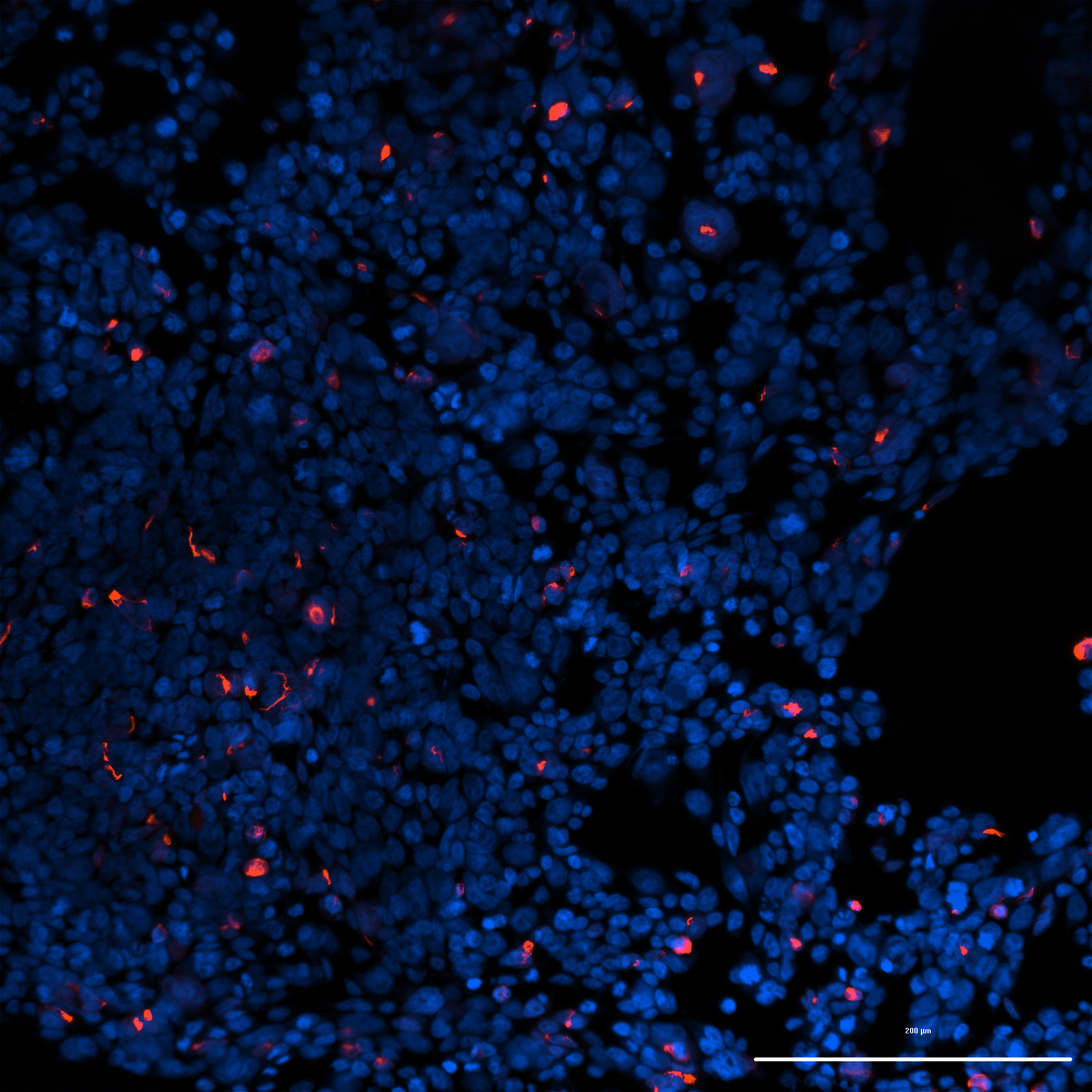

Supplement: Supplementary file 3 — Source data Fig. 3 [file 44318_2025_626_MOESM3_ESM.zip › Source data for Figure 3/3D/WT_IFI16 PYD_20x_2m.png]

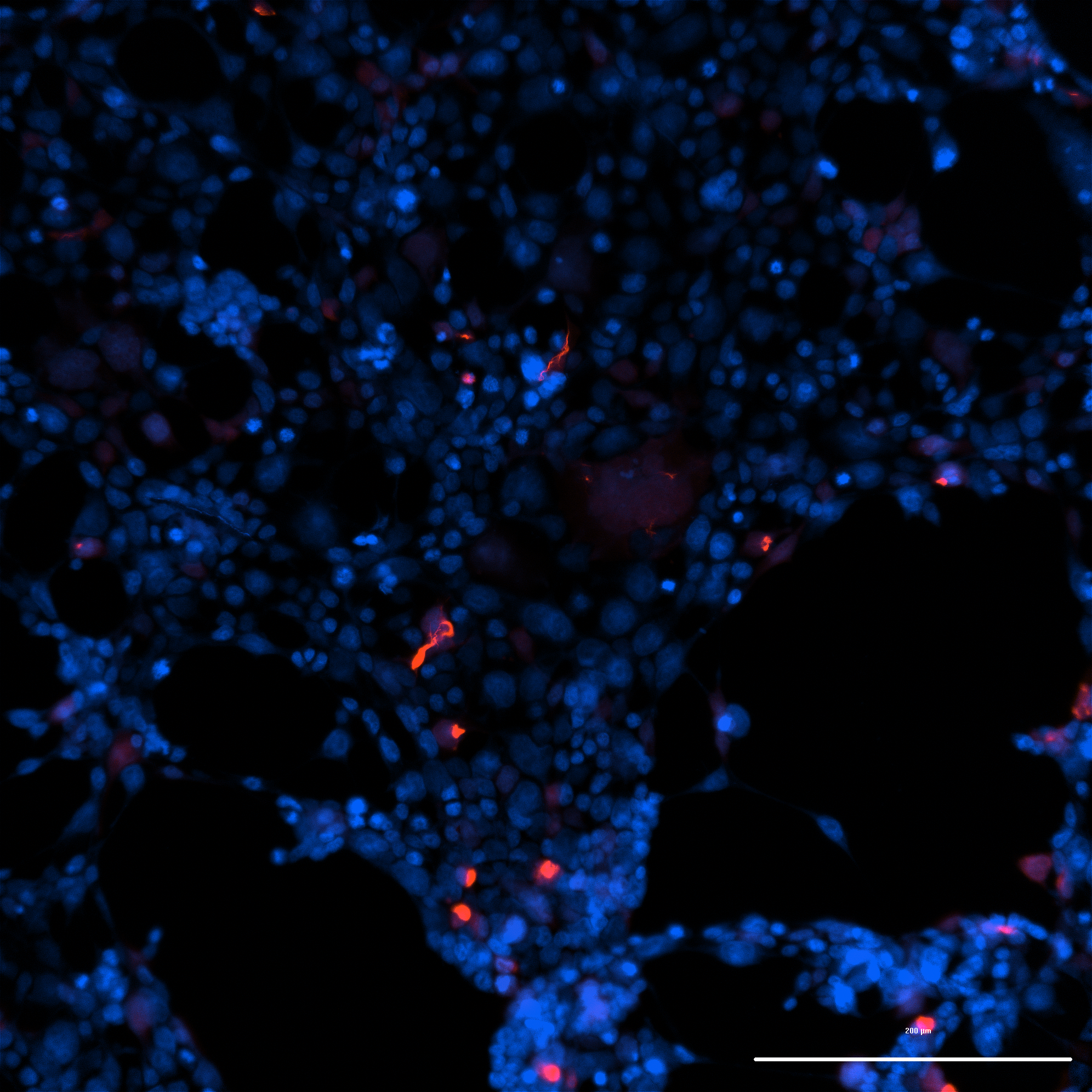

Supplement: Supplementary file 3 — Source data Fig. 3 [file 44318_2025_626_MOESM3_ESM.zip › Source data for Figure 3/3D/M39K_IFI16 PYD_20x_19m.png]

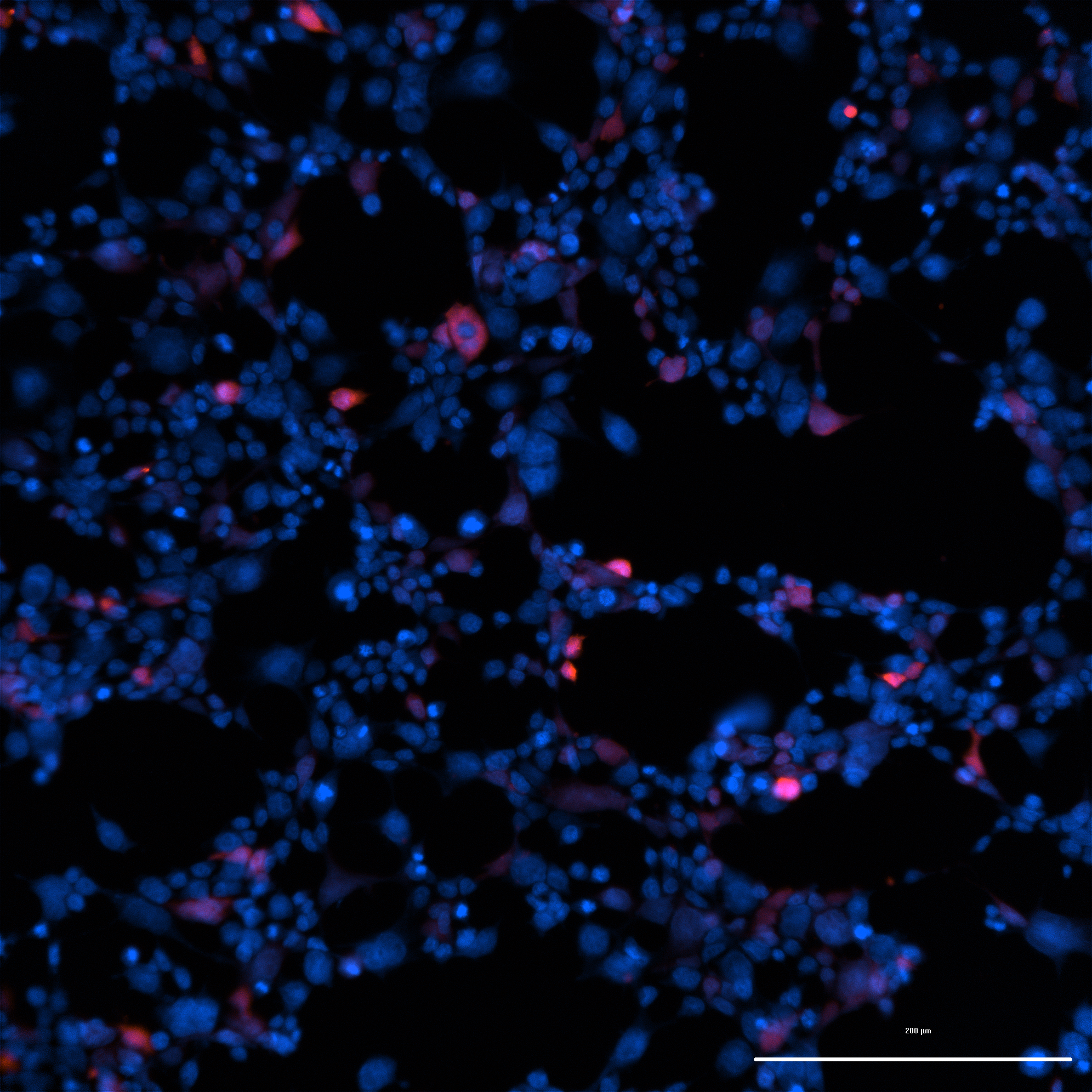

Supplement: Supplementary file 3 — Source data Fig. 3 [file 44318_2025_626_MOESM3_ESM.zip › Source data for Figure 3/3D/P73N_IFI16 PYD_20x_9m.png]

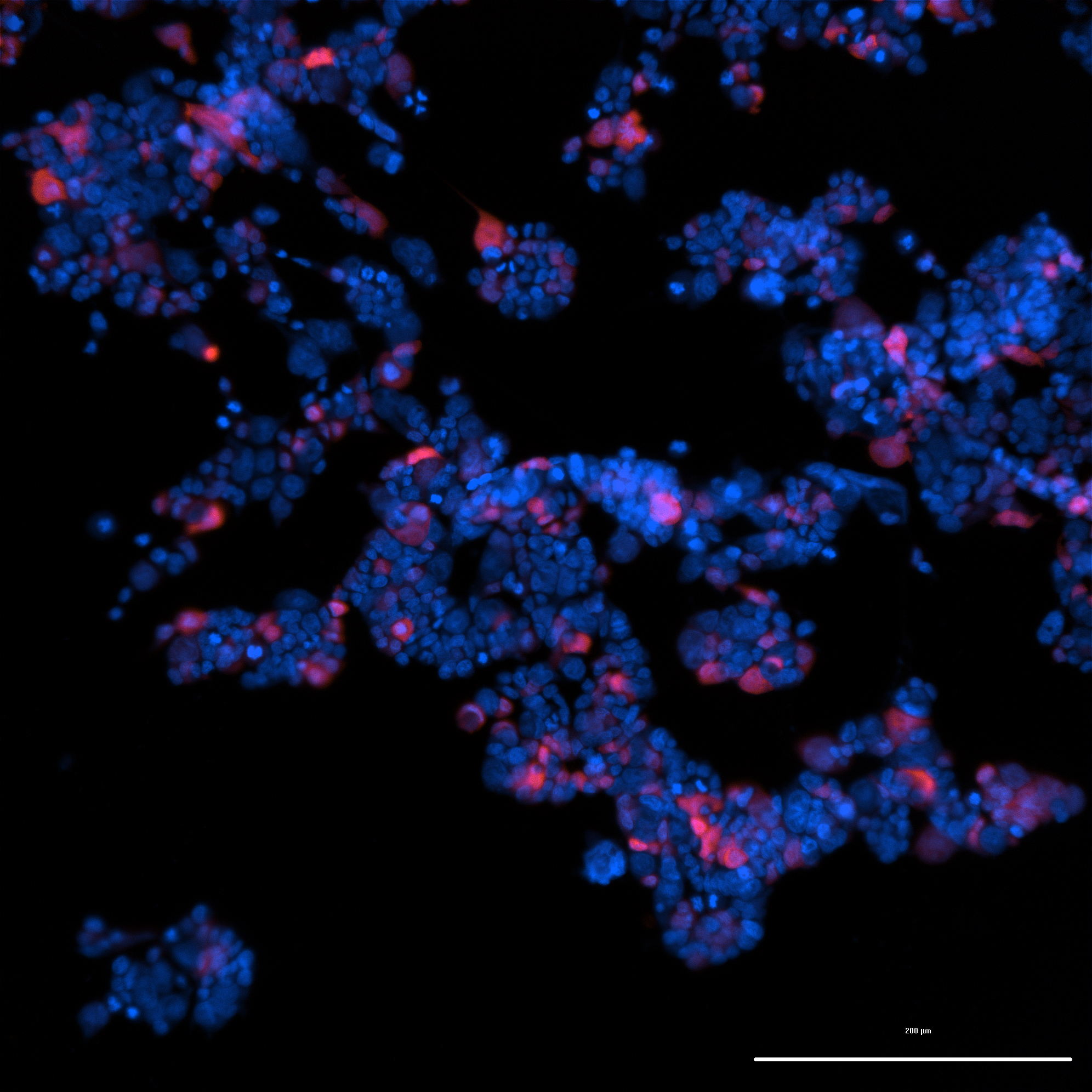

Supplement: Supplementary file 3 — Source data Fig. 3 [file 44318_2025_626_MOESM3_ESM.zip › Source data for Figure 3/3D/R40L_IFI16 PYD_20x_6m.png]

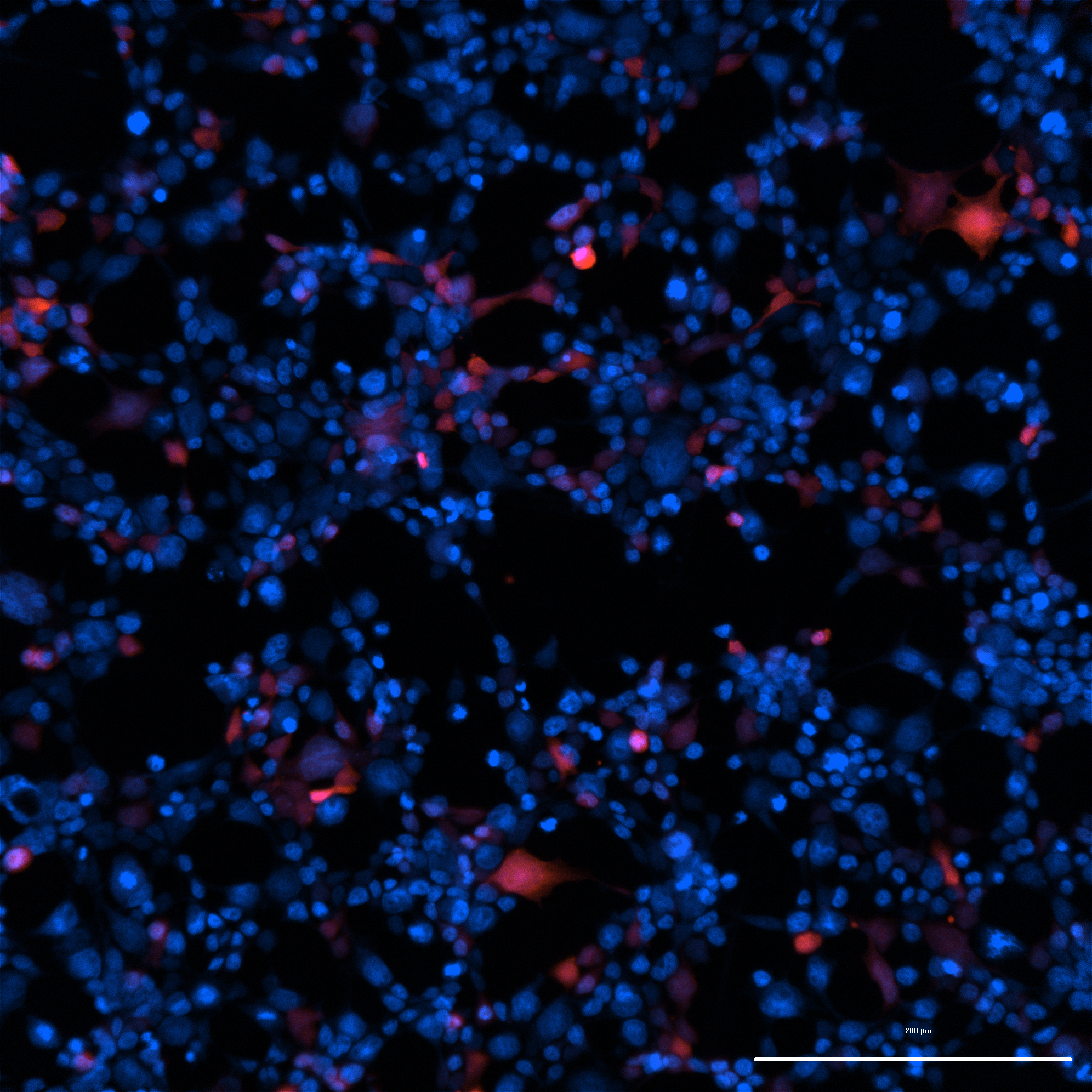

Supplement: Supplementary file 3 — Source data Fig. 3 [file 44318_2025_626_MOESM3_ESM.zip › Source data for Figure 3/3D/K12T_IFI16 PYD_20x_2m.png]

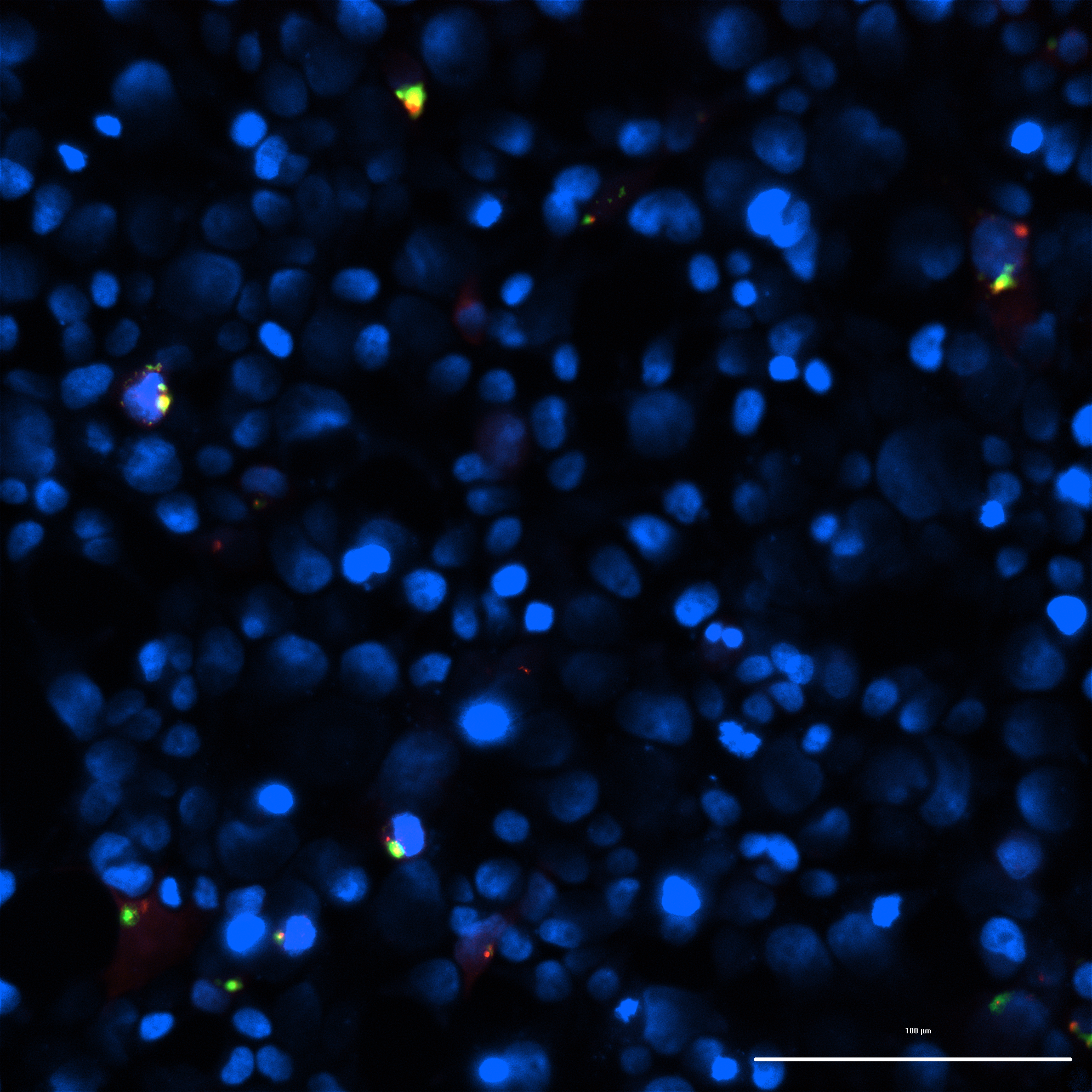

Supplement: Supplementary file 4 — Source data Fig. 4 [file 44318_2025_626_MOESM4_ESM.zip › Source data for Figure 4/4E/ASC FL eGFP plus AIM2 FL mCherry_40x_6m.png]

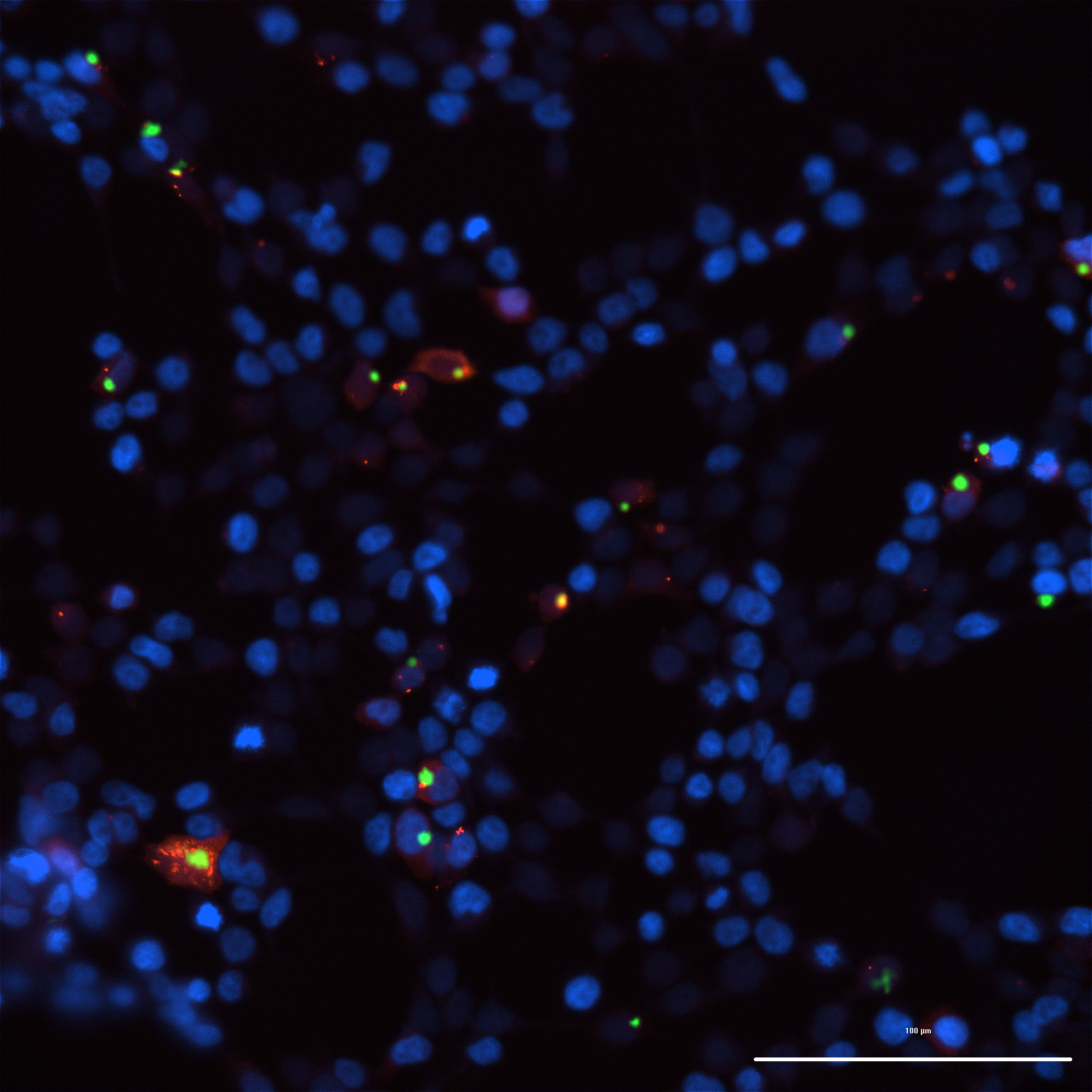

Supplement: Supplementary file 4 — Source data Fig. 4 [file 44318_2025_626_MOESM4_ESM.zip › Source data for Figure 4/4E/ASC FL eGFP plus IFI16 FL K128Q mCherry_40x_6m.png]

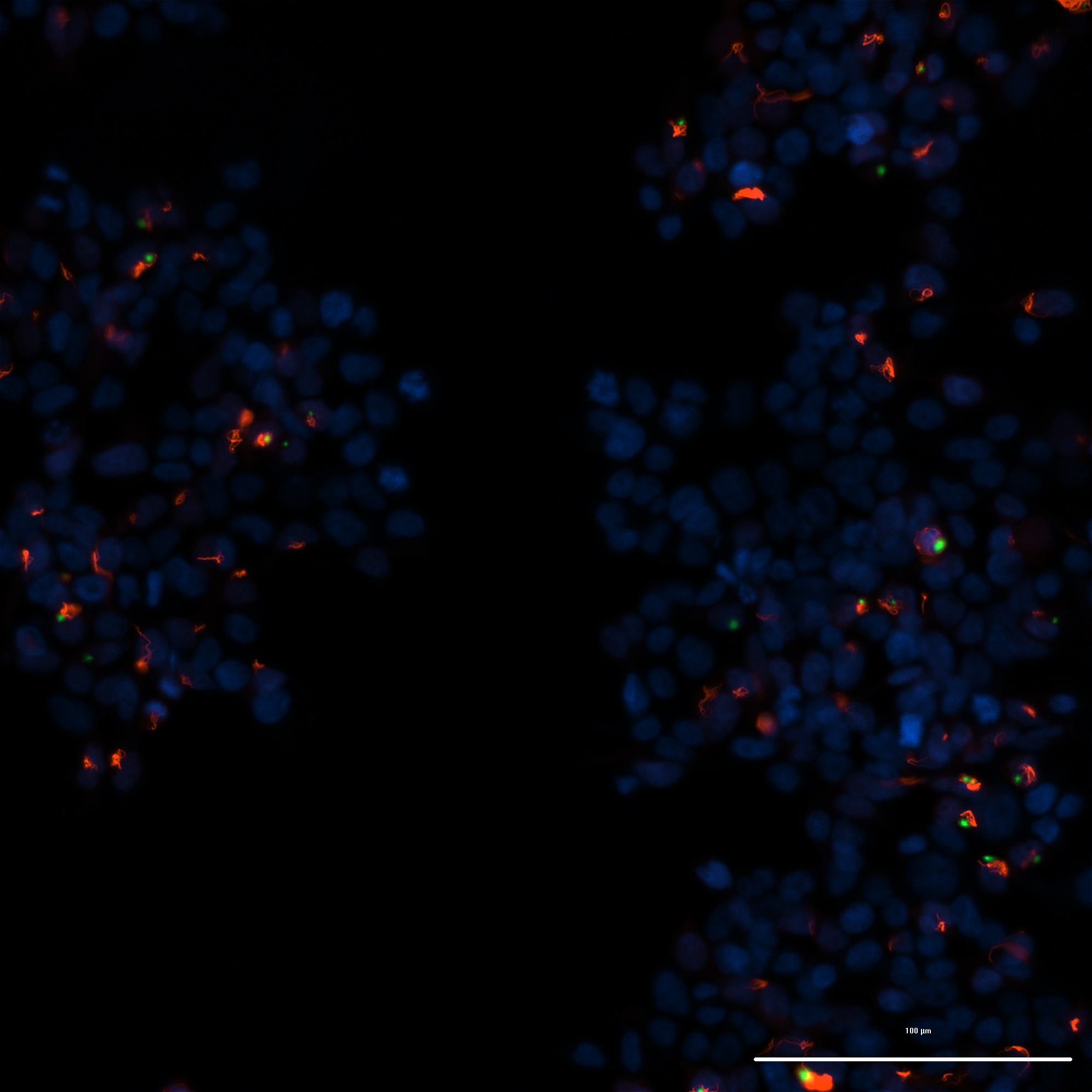

Supplement: Supplementary file 4 — Source data Fig. 4 [file 44318_2025_626_MOESM4_ESM.zip › Source data for Figure 4/4E/ASC FL eGFP plus IFI16PYD mCherry 40x_4m.png]

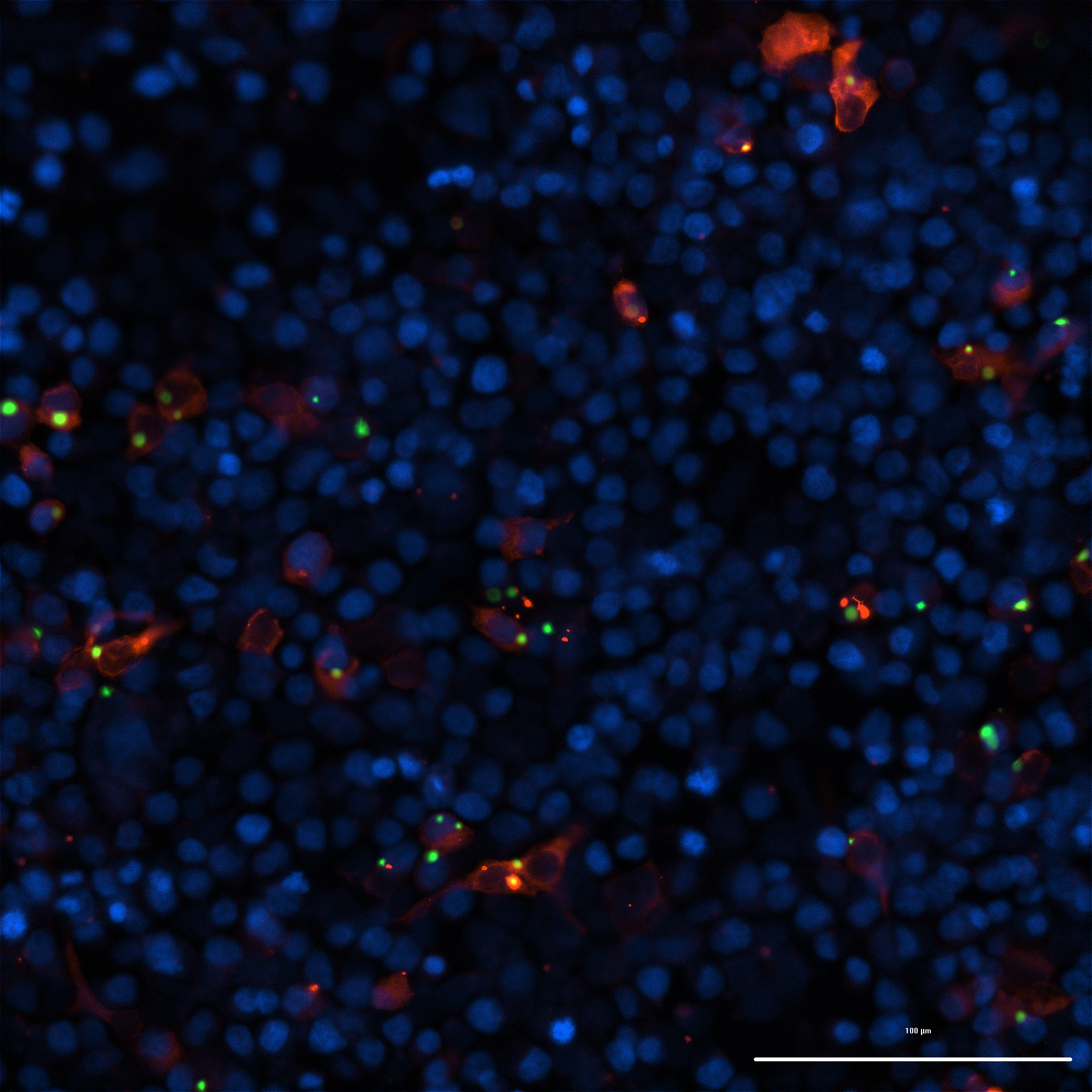

Supplement: Supplementary file 4 — Source data Fig. 4 [file 44318_2025_626_MOESM4_ESM.zip › Source data for Figure 4/4E/ASC FL eGFP plus IFI16 FL delMotif1 mCherry_40x_8m.png]

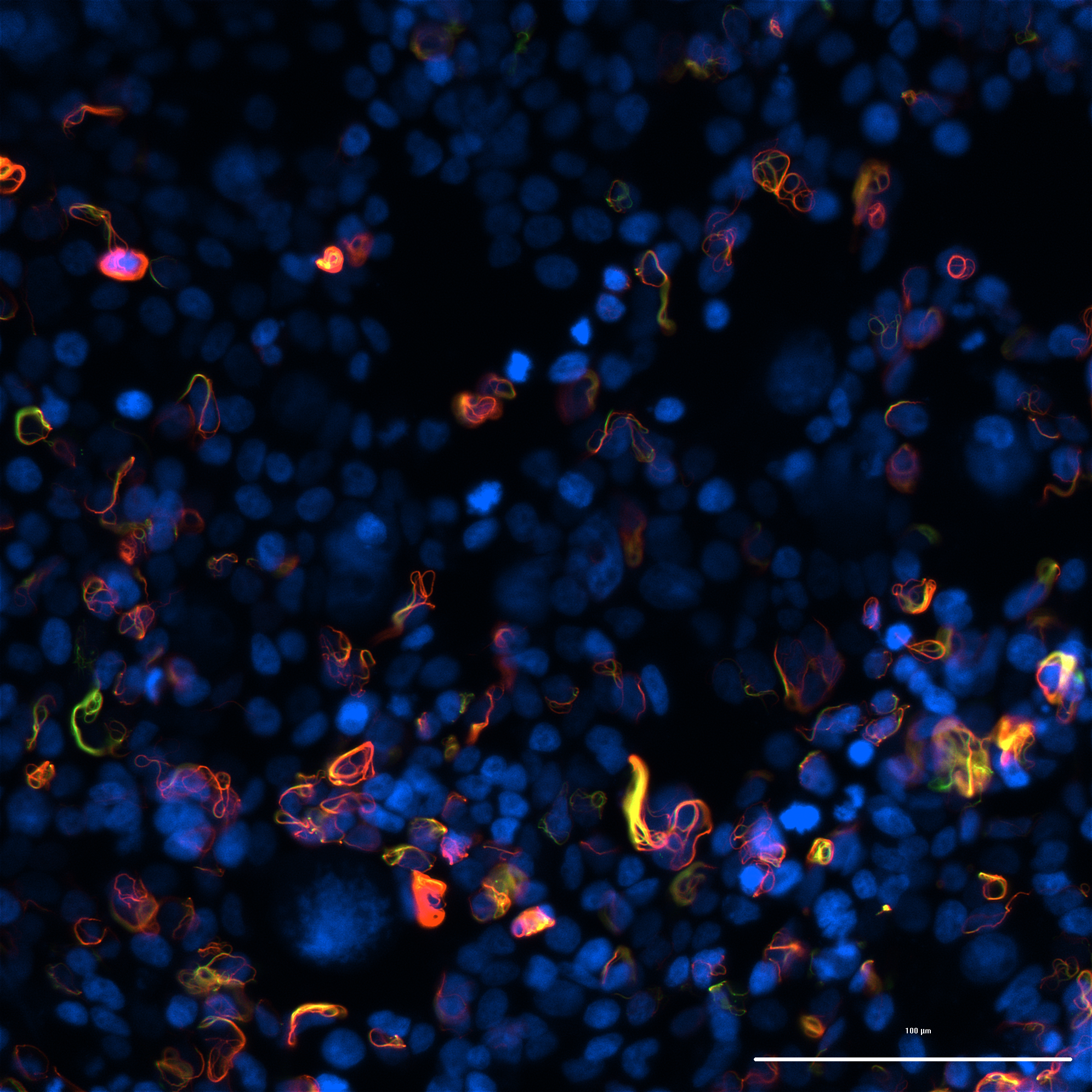

Supplement: Supplementary file 4 — Source data Fig. 4 [file 44318_2025_626_MOESM4_ESM.zip › Source data for Figure 4/4D/300AIM2 PYD eGFP plus 300ASC PYD mCherry_40x_5m.png]

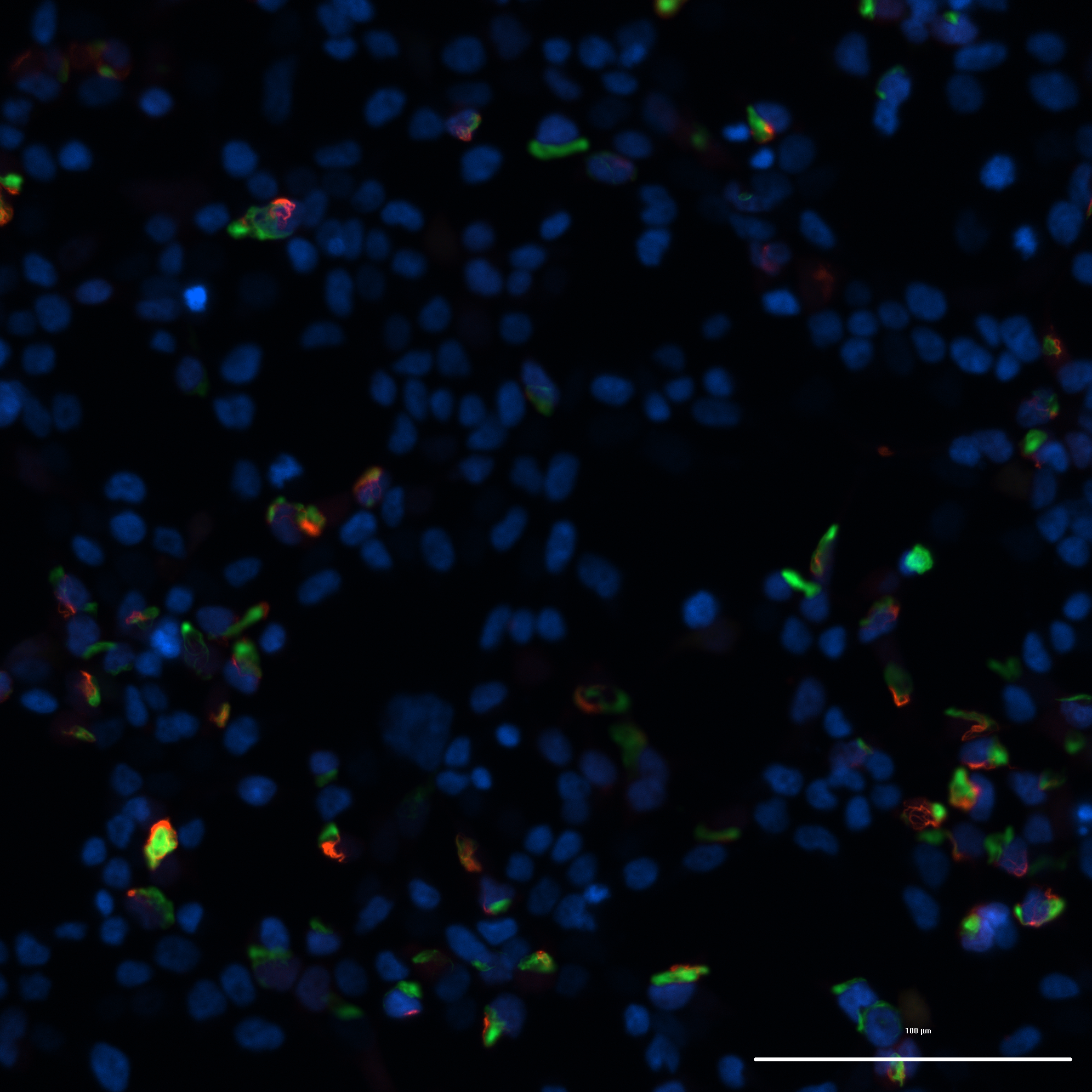

Supplement: Supplementary file 4 — Source data Fig. 4 [file 44318_2025_626_MOESM4_ESM.zip › Source data for Figure 4/4D/ASC PYD eGFP plus IFI16PYD mCherry_40x_1m.png]

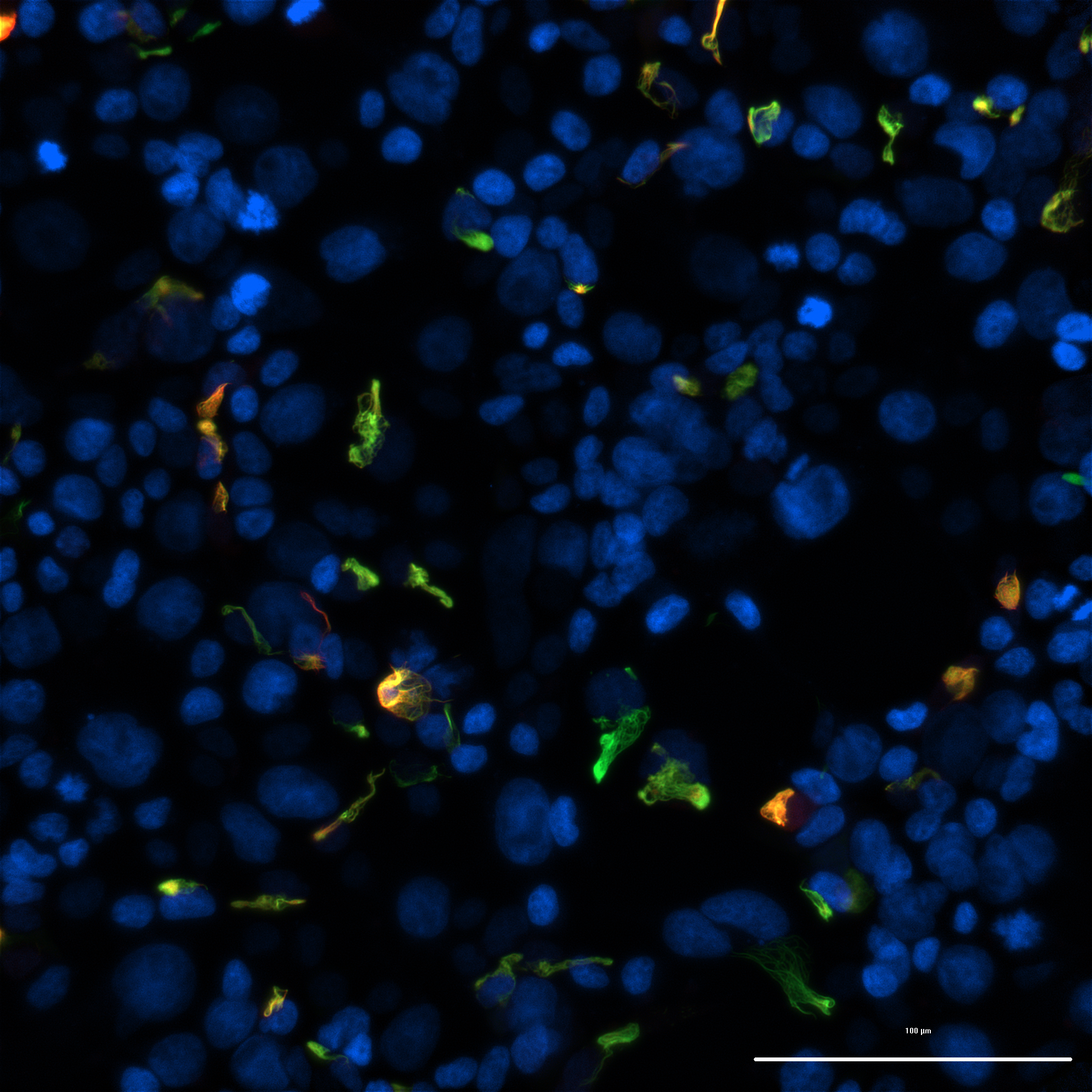

Supplement: Supplementary file 4 — Source data Fig. 4 [file 44318_2025_626_MOESM4_ESM.zip › Source data for Figure 4/4D/ASC-PYD eGFP plus NLRP6 PYD mCherry_40x_9m.png]

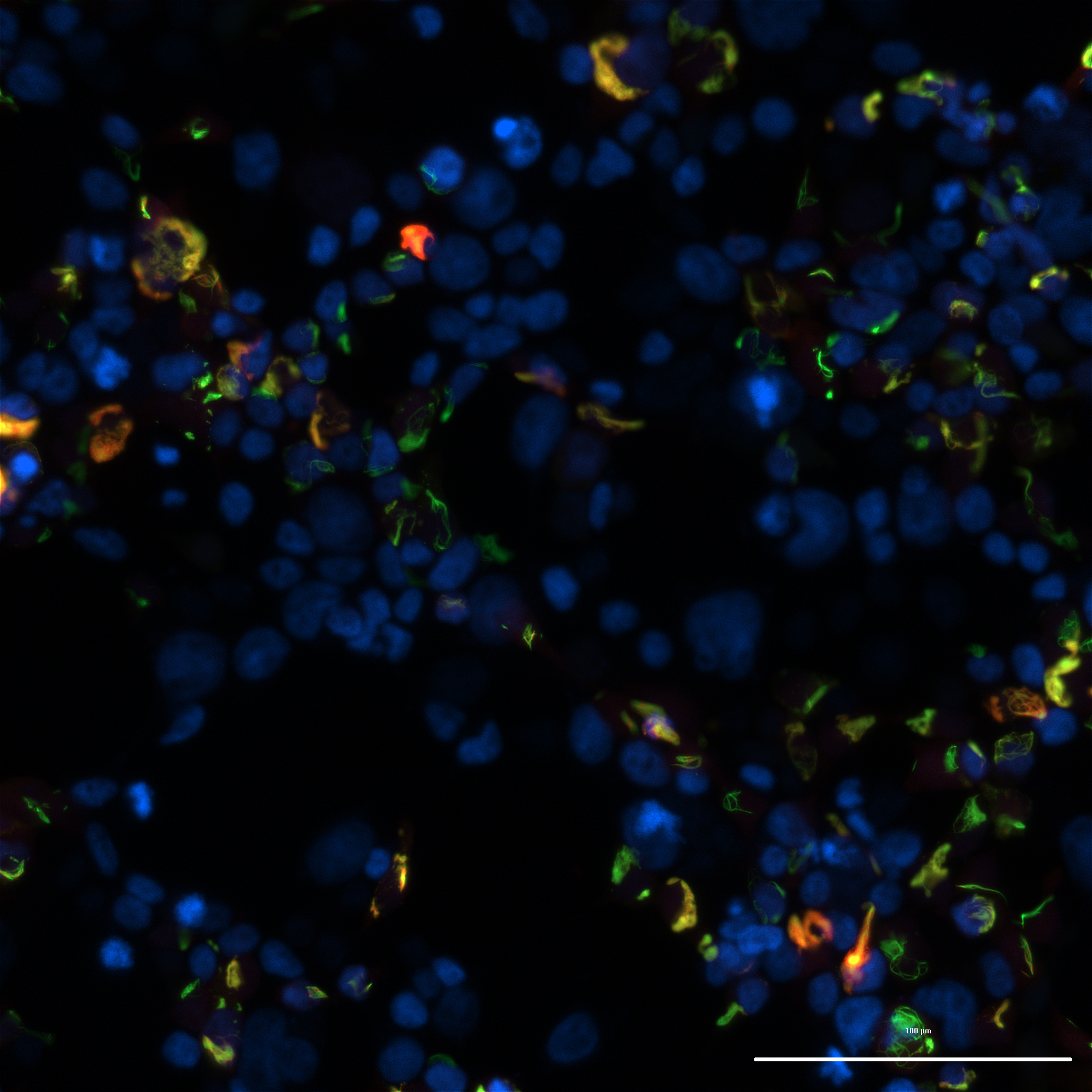

Supplement: Supplementary file 4 — Source data Fig. 4 [file 44318_2025_626_MOESM4_ESM.zip › Source data for Figure 4/4D/ASC PYD-eGFP plus NLRP3PYD-mCherry_40x_1m.png]
