# Supplementary material for: Structural insights into the atypical filament assembly of pyrin domain-containing IFI16
Source: EMBO J. 2025 Nov 5;44(24):7702–20. doi: 10.1038/s44318-025-00626-7 (PMC12705702; doi:10.1038/s44318-025-00626-7)
Supplement: Supplementary file 5 — Expanded View Figures [file 44318_2025_626_MOESM5_ESM.pdf]

Expanded View Figures

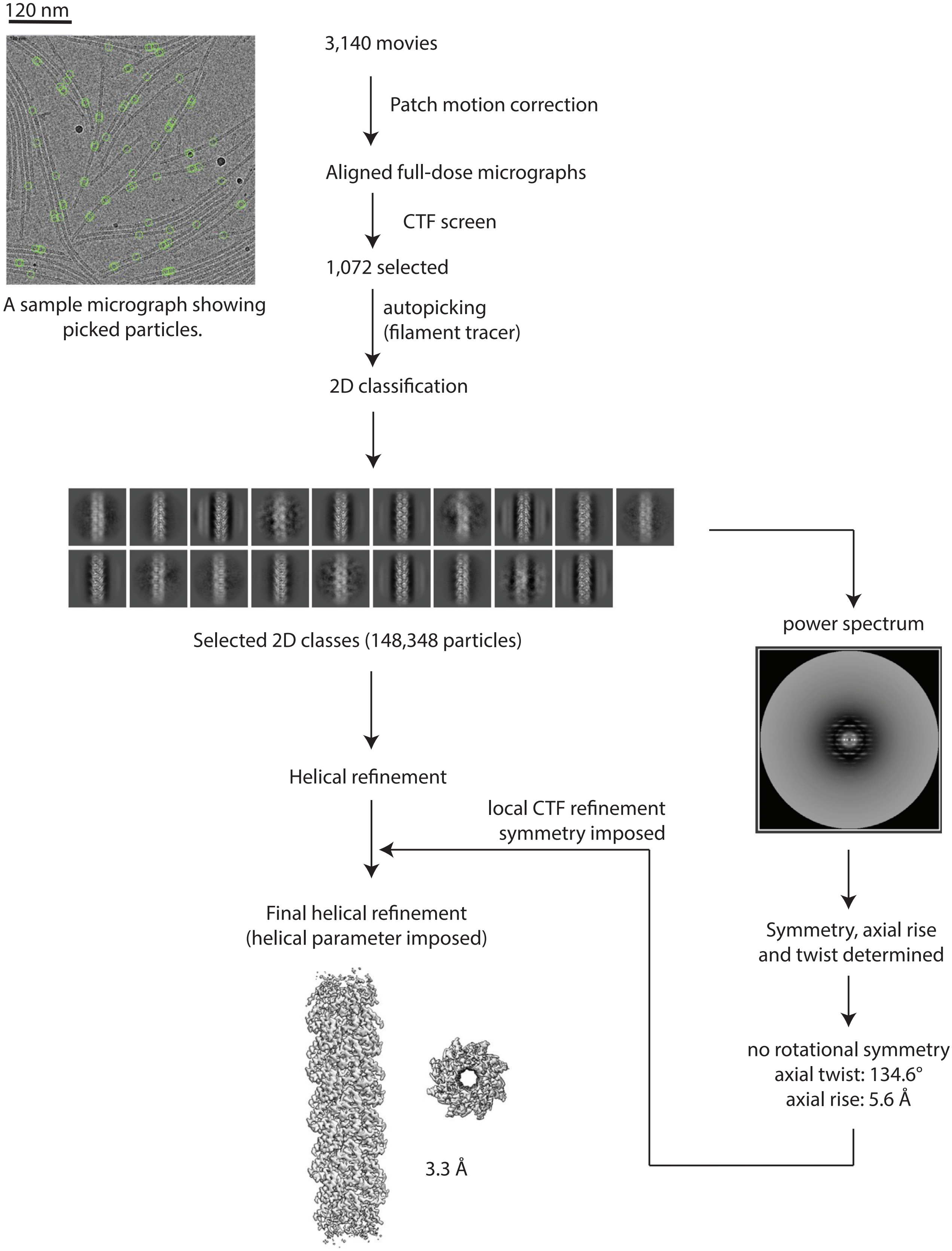

Figure EV1. Cryo-EM image processing workflow for $IFI16^{PYD}$ data detailing the processing from collected micrographs to the final map.

© The Author(s)

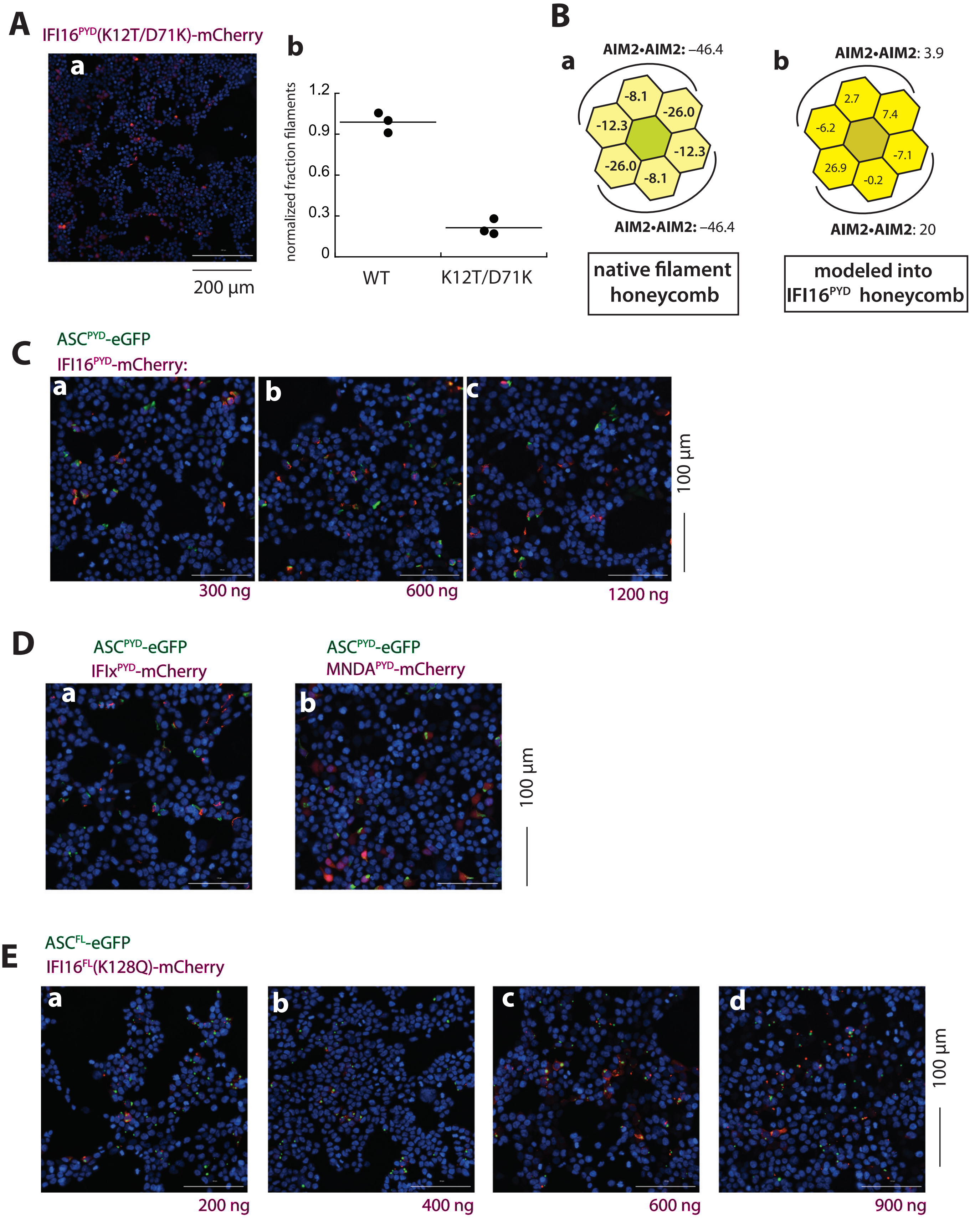
A
IFI16PYD(K12T/D71K)-mCherry
a
200 µm
b
normalized fraction filaments
1.2
0.9
0.6
0.3
0
WT
K12T/D71K
B
a
AIM2•AIM2: –46.4
-8.1
-26.0
-12.3
-12.3
-26.0
-8.1
AIM2•AIM2: –46.4
native filament honeycomb
b
AIM2•AIM2: 3.9
2.7
7.4
-6.2
-7.1
26.9
-0.2
AIM2•AIM2: 20
modeled into IFI16PYD honeycomb
C
ASCPYD-eGFP
IFI16PYD-mCherry:
a
b
c
100 µm
300 ng
600 ng
1200 ng
D
ASCPYD-eGFP
IFIxPYD-mCherry
a
ASCPYD-eGFP
MNDAPYD-mCherry
b
100 µm
E
ASCFL-eGFP
IFI16FL(K128Q)-mCherry
a
b
c
d
100 µm
200 ng
400 ng
600 ng
900 ng

 © The Author(s)

Figure EV2. Cellular and *Rosetta* analyses of IFI16^{PYD} filament assembly and its interaction with ASC.

(**A**) (**a**) Fluorescence microscope image of HEK293T cells transfected with mCherry-tagged K12T/D71K-IFI16^{PYD} (1200 ng) Blue: Hoechst. (**B**) A plot showing the relative amount of filaments formed by indicated IFI16^{PYD} variants in HEK293T cells. $n \geq 3$ biological replicates. (**b**) The *reu*s of the AIM2^{PYD} honeycombs modeled when using (**A**) the native filament (PDB: 7k3r) vs. (**B**) that of IFI16^{PYD}. (**C**) Fluorescence microscope images of HEK293T cells co-transfected with eGFP-tagged ASC^{PYD} (600 ng) and the indicated amount of mCherry-tagged IFI16^{PYD}. Blue: Hoechst. $n \geq 3$ biological replicates. (**D**) Fluorescence microscope images of HEK293T cells co-transfected with eGFP-tagged ASC^{PYD} (600 ng) and mCherry-tagged IFIx^{PYD} and MNDA^{PYD} (both 600 ng) Blue: Hoechst. n ≥ 3 biological replicates. (**E**) Fluorescence microscope images of HEK293T cells co-transfected with eGFP-tagged ASC^{FL} (600 ng) and the indicated amount of mCherry-tagged K128Q-IFI16^{FL}. Blue: Hoechst. $n \geq 3$ biological replicates.

© The Author(s)

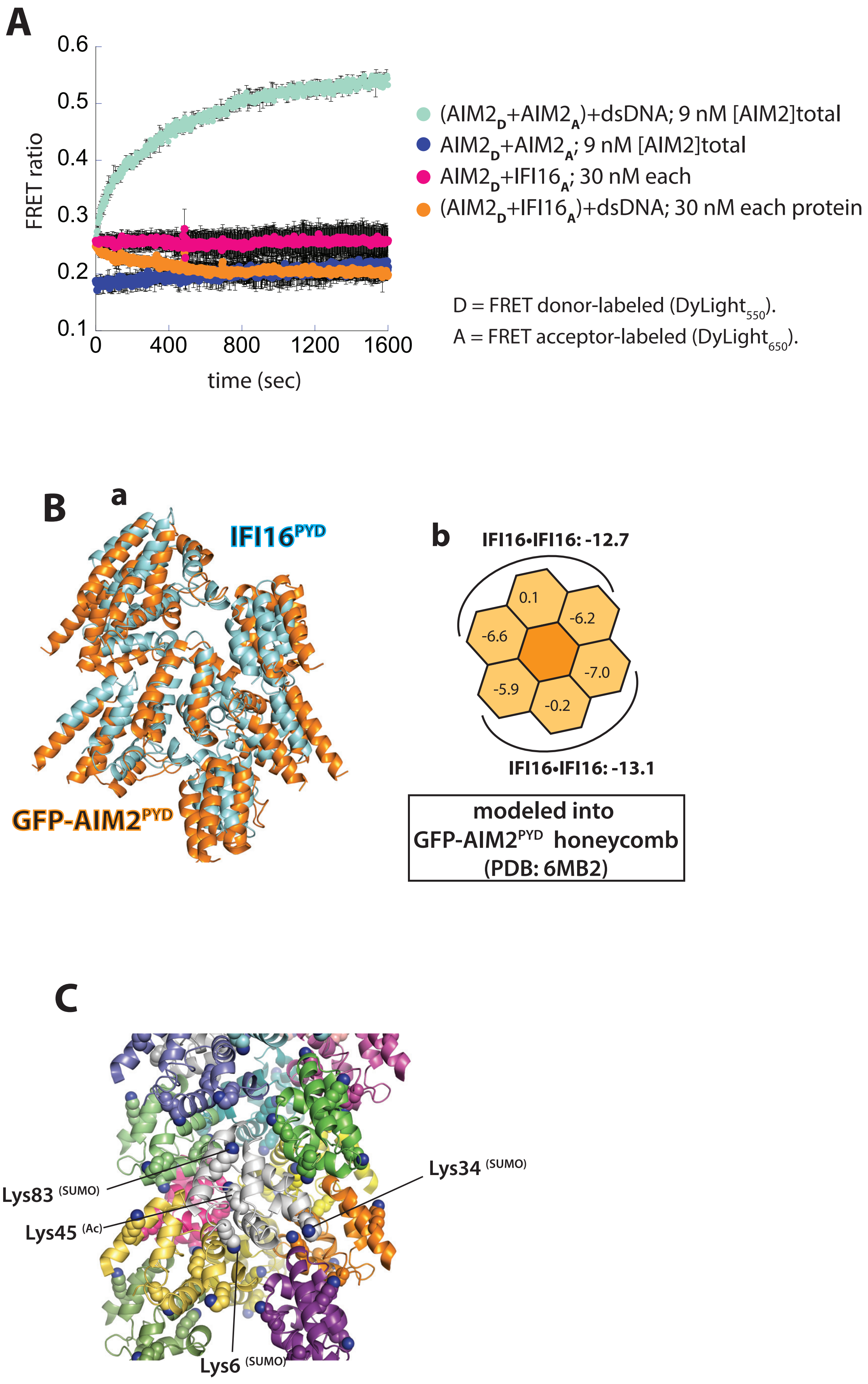
A
FRET ratio
0.6
0.5
0.4
0.3
0.2
0.1
0
400
800
1200
1600
time (sec)
$(AIM2_D+AIM2_A)$+dsDNA; 9 nM [AIM2]total
$AIM2_D+AIM2_A$; 9 nM [AIM2]total
$AIM2_D+IFI16_A$; 30 nM each
$(AIM2_D+IFI16_A)$+dsDNA; 30 nM each protein
D = FRET donor-labeled ($DyLight_{550}$).
A = FRET acceptor-labeled ($DyLight_{650}$).
B
a
$IFI16^{PYD}$
$GFP\text{-}AIM2^{PYD}$
b
IFI16•IFI16: -12.7
0.1
-6.2
-6.6
-7.0
-5.9
-0.2
IFI16•IFI16: -13.1
modeled into
$GFP\text{-}AIM2^{PYD}$ honeycomb
(PDB: 6MB2)
C
Lys83 (SUMO)
Lys45 (Ac)
Lys34 (SUMO)
Lys6 (SUMO)

 © The Author(s)

Figure EV3. IFI16 does not interact iwth AIM2.

(**A**) A plot showing changes in the FRET ratio between indicated labeled proteins in the presence and absence of 600-bp dsDNA (10 μg/ml). Shown is the average of three independent experiments. Error bars = standard deviations. $n \geq 3$ biological replicates. (**B**) (**a**) An overlay between GFP-AIM2^{PYD} (PDB ID: 6mb2) and IFI16^{PYD} filaments. (**B**) The *reu*s of the IFI16^{PYD} honeycomb when modeled using the GFP-AIM2^{PYD} filament. (**C**) A cartoon showing the locations of putative (SUMOylation) and identified (acetylation) PTM sites on IFI16^{PYD}.
